# Identification of biomarkers in nonalcoholic fatty liver disease: A machine learning method and experimental study

**DOI:** 10.3389/fgene.2022.1020899

**Published:** 2022-11-07

**Authors:** Na Han, Juan He, Lixin Shi, Miao Zhang, Jing Zheng, Yuanshuo Fan

**Affiliations:** ^1^ Department of Endocrinology, The Affiliated Hospital of Guizhou Medical University, Guiyang, China; ^2^ Department of Endocrinology, Guizhou Provincial People’s Hospital, Guiyang, China

**Keywords:** NAFLD, machine learning, biomarkers, bioinformactics, immune infiltration

## Abstract

Nonalcoholic fatty liver disease (NAFLD) has become the most common chronic liver disease. However, the early diagnosis of NAFLD is challenging. Thus, the purpose of this study was to identify diagnostic biomarkers of NAFLD using machine learning algorithms. Differentially expressed genes between NAFLD and normal samples were identified separately from the GEO database. The key DEGs were selected through a protein‒protein interaction network, and their biological functions were analysed. Next, three machine learning algorithms were selected to construct models of NAFLD separately, and the model with the smallest sample residual was determined to be the best model. Then, logistic regression analysis was used to judge the accuracy of the five genes in predicting the risk of NAFLD. A single-sample gene set enrichment analysis algorithm was used to evaluate the immune cell infiltration of NAFLD, and the correlation between diagnostic biomarkers and immune cell infiltration was analysed. Finally, 10 pairs of peripheral blood samples from NAFLD patients and normal controls were collected for RNA isolation and quantitative real-time polymerase chain reaction for validation. Taken together, CEBPD, H4C11, CEBPB, GATA3, and KLF4 were identified as diagnostic biomarkers of NAFLD by machine learning algorithms and were related to immune cell infiltration in NAFLD. These key genes provide novel insights into the mechanisms and treatment of patients with NAFLD.

## Introduction

Nonalcoholic fatty liver disease (NAFLD) is the most common chronic disease of the liver, and the global prevalence of NAFLD among adults is estimated to be 23%–25% (Huang et al., 2021) (Estes et al., 2018). A recent meta-analysis showed an unexpected rapid increase in the burden of NAFLD in China over the past 10 years, with a prevalence of 29.2% (Zhou et al., 2020). NAFLD is a clinicopathological entity that encompasses a wide range of liver disease spectra (Calzadilla Bertot and Adams, 2016). The majority of people living with NAFLD have isolated steatosis (nonalcoholic fatty liver, NAFL), and a smaller proportion develop nonalcoholic steatohepatitis (NASH), with increasing hepatic fibrosis eventually leading to cirrhosis, liver cancer, end-stage liver disease and death (Lazarus et al., 2022a). Moreover, NAFLD increases the risk of other metabolic diseases, such as diabetes, cardiovascular disease, and chronic kidney disease.

Liver biopsy is the gold standard for diagnosing NAFLD. However, due to its invasiveness, potential bleeding risk, and large sampling error caused by the uneven distribution of liver parenchymal lesions, liver biopsy cannot be well applied in clinical practice (Ratziu et al., 2005). For these reasons, the diagnosis and treatment of NAFLD are usually delayed. Early discovery of NAFLD and mainly of NASH brings a great advantage because there are many drugs on the pipeline that are good candidates to cure this very common disease, as evident in various recent papers (Negi et al., 2022). Therefore, exploring accurate, noninvasive biomarkers for diagnosing and staging NAFLD is critical for reducing the need for an invasive liver biopsy and to identify patients who are at high risk of hepatic and cardio-metabolic complications as early as possible. Moreover, biomarkers may assist us in investigating the mechanisms of NAFLD pathogenesis.

Machine learning is a branch of artificial intelligence that allows researchers to use complex data and develop self-trained strategies to predict the characteristics of new samples (Lynch and Liston, 2018). The algorithms have been applied in many clinical fields, including disease prediction, diagnosis, prognosis, and drug discovery (Qin et al., 2022). For example, they have been applied for breast cancer (Hanis et al., 2022), ovarian cancer (Lu et al., 2020), colorectal cancer (Zhang et al., 2022), hepatocellular carcinoma (Gupta et al., 2021), cholangiocarcinoma (Liu et al., 2021), nonfunctioning pituitary adenoma (Fang et al., 2021), and nasopharyngeal carcinoma (Zhang et al., 2017). Therefore, in the context of machine learning methods, we reviewed various research studies with novel biomarkers for the diagnosis of NAFLD.

In our study, NAFLD and normal sample datasets were systematically retrieved and obtained from the Gene Expression Omnibus (GEO) database, and differentially expressed genes (DEGs) were screened out through the robust rank aggregation (RRA) method. To explore the DEG function and main metabolic and signal transduction pathways, we used functional enrichment and protein‒protein interaction (PPI) analysis. We modelled three machine learning models to obtain the diagnostic biomarkers (Han et al., 2015). The predictive ability of the diagnostic biomarkers for NAFLD was further evaluated by a nomogram. Inflammation is closely associated with immune cells of the liver infiltration (Nati et al., 2022), so we further analysed biomarkers for screening differences in the infiltration of immune cells. Meanwhile, we searched diagnostic biomarkers from the Drug Gene Interaction Database (DGIdb) to obtain potential drugs that could treat NAFLD.

## Materials and methods

### Data collection

The messenger RNA (mRNA) expression matrix and the related clinical information of NAFLD and normal samples in the GSE135251 and GSE126848 datasets were obtained from the GEO database (https://www.ncbi.nlm.nih.gov/geo/). The GSE135251 dataset contains 206 NAFLD samples and 10 normal samples (Govaere et al., 2020). The GSE126848 dataset included 15 NAFLD samples and 14 normal samples (Suppli et al., 2019). The sequencing platform of both datasets was a GPL18573 Illumina NextSeq 500 (*Homo sapiens*).

### Identification of differentially expressed genes

The DEGs between NAFLD and normal samples in the GSE126848 and GSE135251 datasets were selected by the “limma” R package (version 3.46.0). The screening conditions were as follows: log2|FC| > 1, *p* < 0.05. The robust rank aggregation (RRA) method can minimize the deviation and error between two datasets and combine them into independent datasets (Kolde et al., 2012). Therefore, the upregulated and downregulated genes in the two datasets were ranked by RRA analysis using the “robustrankaggre” (version 1.1) R package, and Bonferroni correction was performed to finally obtain the optimal DEGs.

### Functional enrichment analysis and interaction of key differentially expressed genes

The protein interaction among key DEGs was explored by the search tool for the retrieval of interacting genes/proteins database (STRING, https://www.string-db.org/), and then the PPI network was constructed by Cyto-scape (version 3.8.2), with a confidence interval of 0.4. At the same time, the m-code plug-in was used to find the key modules and DEGs in the PPI network by setting degree cut-off = 2, node score cut-off = 0.2, k-core = 2, max, depth = 100.

To further explore the targeted pathways and functions of key DEGs, the “cluster-Profiler” R package (Version 3.18.0) was used to conduct Gene Ontology (GO) and Kyoto Encyclopedia of Genes and Genomes (KEGG) enrichment analyses. *p* < 0.05 and a count >2 were considered significant enrichment. In addition, the “enrich-Plot” R package (Version 1.10.2) and “ggplot2” R package (Version 3.3.3) were used to visualize the enrichment results.

### Machine learning screening for diagnostic biomarkers

Based on the expression levels of key DEGs and the grouping information of the samples, in which the sample grouping was used as the response variable and key DEGs were used as the explanatory variable, the “caret” R package (version 6.0-86) was used to build three models: RF, SVM, and GLM. Then, the explain function of the “dalex” R package (version 2.3.0) was used to interpret and analyse the three models, the plot function was used to visualize the performance distribution of the models, and a cumulative residual distribution map and box plot distribution map were drawn to obtain the optimal model. Moreover, the relative importance of different variables in different models for model prediction was analysed. The key DEGs that had a great influence on the predicted value of the response variable were selected as diagnostic markers.

### Nomogram of diagnostic biomarkers and their validation

We further constructed a nomogram through the “rms” R package based on the diagnostic biomarkers to facilitate the clinical judgement of the risk of NAFLD. Then, a calibration curve was drawn to verify the nomogram. In addition, to more intuitively evaluate the clinical effect of the nomogram model, this study used the “rmda” R package to draw a decision curve analysis (DCA) curve and a clinical impact curve on the basis of the DCA curve.

### Immune infiltration analysis

To study the difference in immune infiltration between patients with NAFLD and normal samples, the proportion of 22 immune cells in all samples in the GSE126848 dataset was calculated by the single-sample gene set enrichment analysis (ssGSEA) algorithm using the “GSVA” R package (version 1.38.2). Then, the difference in immune cells between normal and NAFLD samples was compared by the rank-sum test. Finally, the Pearson correlation between diagnostic genes and differential immune cells was analysed.

### Potential drug prediction

Finally, we searched diagnostic biomarkers from the DGIdb (https://dgidb.genome.wustl.edu/) to obtain potential drugs or molecular compounds that can treat NAFLD. Cytoscape (version 3.8.2) software was used to construct the relationship pair network between diagnostic markers and molecular compounds.

### Statistical methods

All statistical analyses were performed with R software 4.0.3. Statistical significance was set at probability values of *p* < 0.05.

### RNA isolation and quantitative real-time polymerase chain reaction

Ten pairs of peripheral blood samples from people with and without fatty liver were collected from The Affiliated Hospital of GuiZhou Medical University. Peripheral blood mononuclear cells were extracted within 4 h after blood collection, and the treated samples were immediately stored at −80°C. All subjects signed informed consent forms. The collection of all samples was approved by the ethics committee of The Affiliated Hospital of Guizhou Medical University (approval No. 2022065K). Total RNA was extracted from the peripheral blood of all samples with TRIzol reagent (cat. 356281) provided by the Ambion company. Then, a Nanodrop 2000fc-3100 (Thermo Fisher Scientific, Waltham, MA, United States) was used to quantify the concentration and purity of the RNA solution. A sweScript RT I First-Strand cDNA Synthesis All-in-One™ First-Strand cDNA Synthesis Kit (CAT-G33330-50) provided by the Service-bio company was used for the reverse transcription reaction. PCR was performed using the 2x Universal Blue SYBR Green qPCR Master Mix (CAT.-G3326-05) kit provided by Service-bio. The PCR conditions were as follows: 95°C predenaturation for 1 min and then 40 cycles. Each cycle included denaturation at 95°C for 20 s, annealing at 55°C for 20 s, and extension at 72°C for 30 s. GAPDH was used as an internal reference for gene detection. The forward primer for GAPDH was “CCC​ATC​ACC​ATC​TTC​CAG​G”. The reverse primer for GAPDH was “CAT​CAC​GCC​ACA​GTT​TCC​C”. The forward primer for CEBPD was “GCCCCCGCCATGTAC”. The reverse primer for CEBPD was “GCCCGCCTTGTGATT”. The forward primer for H4C11 was “GCG​GGG​TGC​TGA​AGG​TGT​T”. The reverse primer for H4C11 was “GCT​TGG​CGT​GCT​CTG​TAT​A”. The forward primer for CEBPB was “TGG​GAC​CCA​GCA​TGT​CTC”. The reverse primer for CEBPB was “CAG​TTC​TTG​CCC​CCG​TAG”. The forward primer for GATA3 was “CAC​CTC​TTC​ACC​TTC​CCG”. The reverse primer for GATA3 was “TTG​CCC​CAC​AGT​TCA​CAC”. The forward primer for KLF4 was “GAG​GAG​CCC​AAG​CCA​AAG”. The reverse primer for KLF4 was “CAG​CCG​TCC​CAG​TCA​CAG”. A *t*-test was used to compare the expression of five biomarkers between patients with NAFLD and the control group. *p* < 0.05 was considered significant.

## Results

### Identification of differentially expressed genes

A total of 9,005 DEGs between NAFLD and normal samples, among which 47 genes were upregulated and 8,958 genes were downregulated in NAFLD samples, were screened in the GSE126848 dataset. In the GSE135251 dataset, 1,489 upregulated and 300 downregulated genes in NAFLD samples compared with normal samples were identified.

**FIGURE 1:**
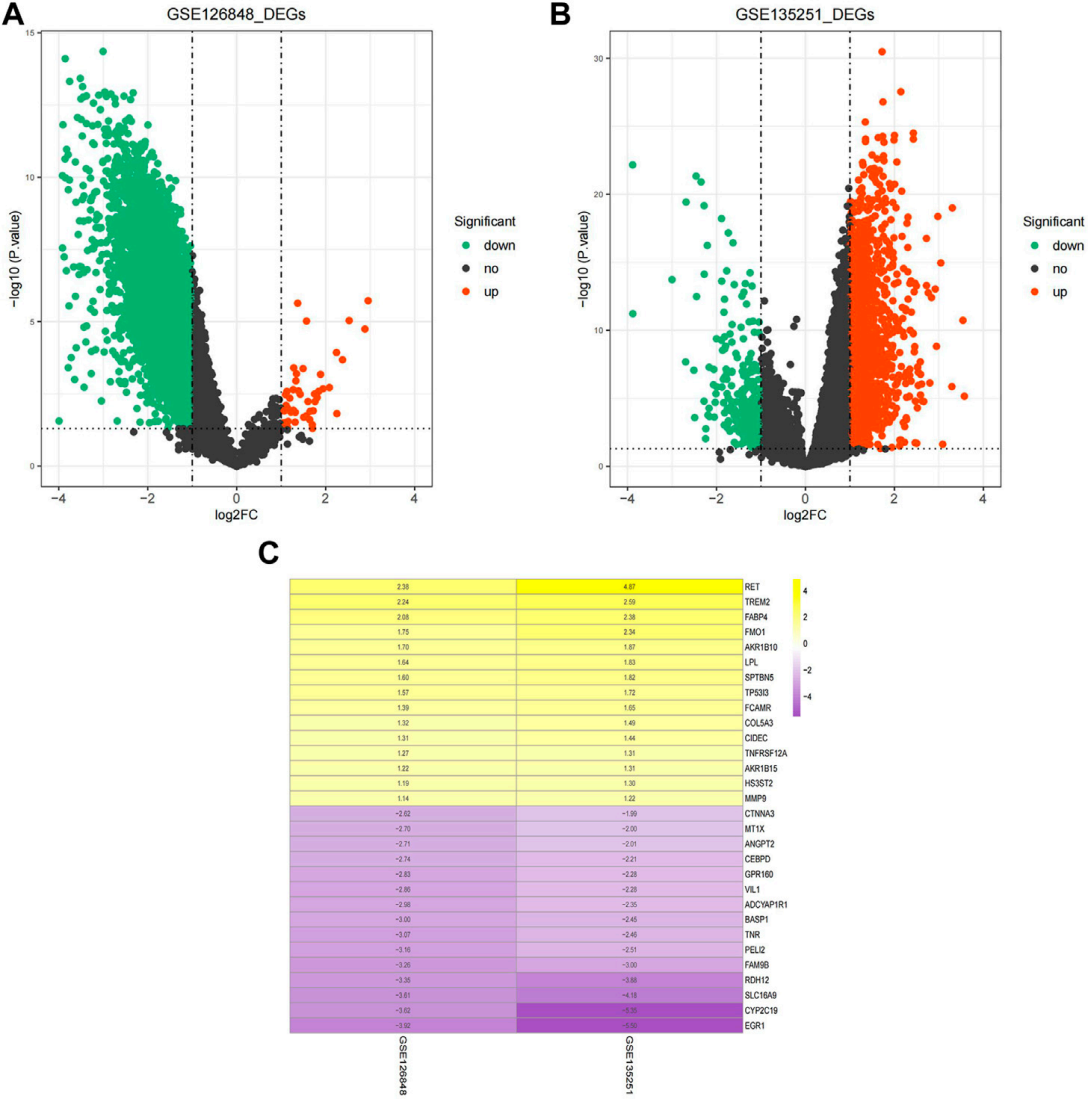
Identification of DEGs. **(A)** Volcano plot of DEGs in the GSE126848 dataset. **(B)** Volcano plot of DEGs in the GSE135251 dataset. The abscissa of log2FC is the fold change (NAFLD/normal), and the ordinate is the credibility-log10 (*p-value*). Each dot in the volcano plot indicates a gene, and the red and green dots indicate significant DEGs. Red dots indicate that gene expression is upregulated in NAFLD samples, and green dots indicate that gene expression is downregulated in NAFLD samples. Dashed lines of abscissa and ordinate indicate the absolute log FC threshold of 1 and the *p-value* threshold of 0.05, respectively. **(C)** Heatmap of upregulated and downregulated DEGs. Yellow indicates upregulated DEGs, purple indicates downregulated DEGs. The darker the colour is, the more significant the upregulation and downregulation.

The volcano plots of the DEGs are shown in Figures 1A,B. The DEGs of the two datasets were integrated and corrected by the RRA method, and a total of 147 key DEGs were obtained (see Supplementary Table S1). A heatmap of the top 15 upregulated and downregulated genes is shown in Figure 1C.

### Functional enrichment analysis and interaction of key differentially expressed genes

**FIGURE 2:**
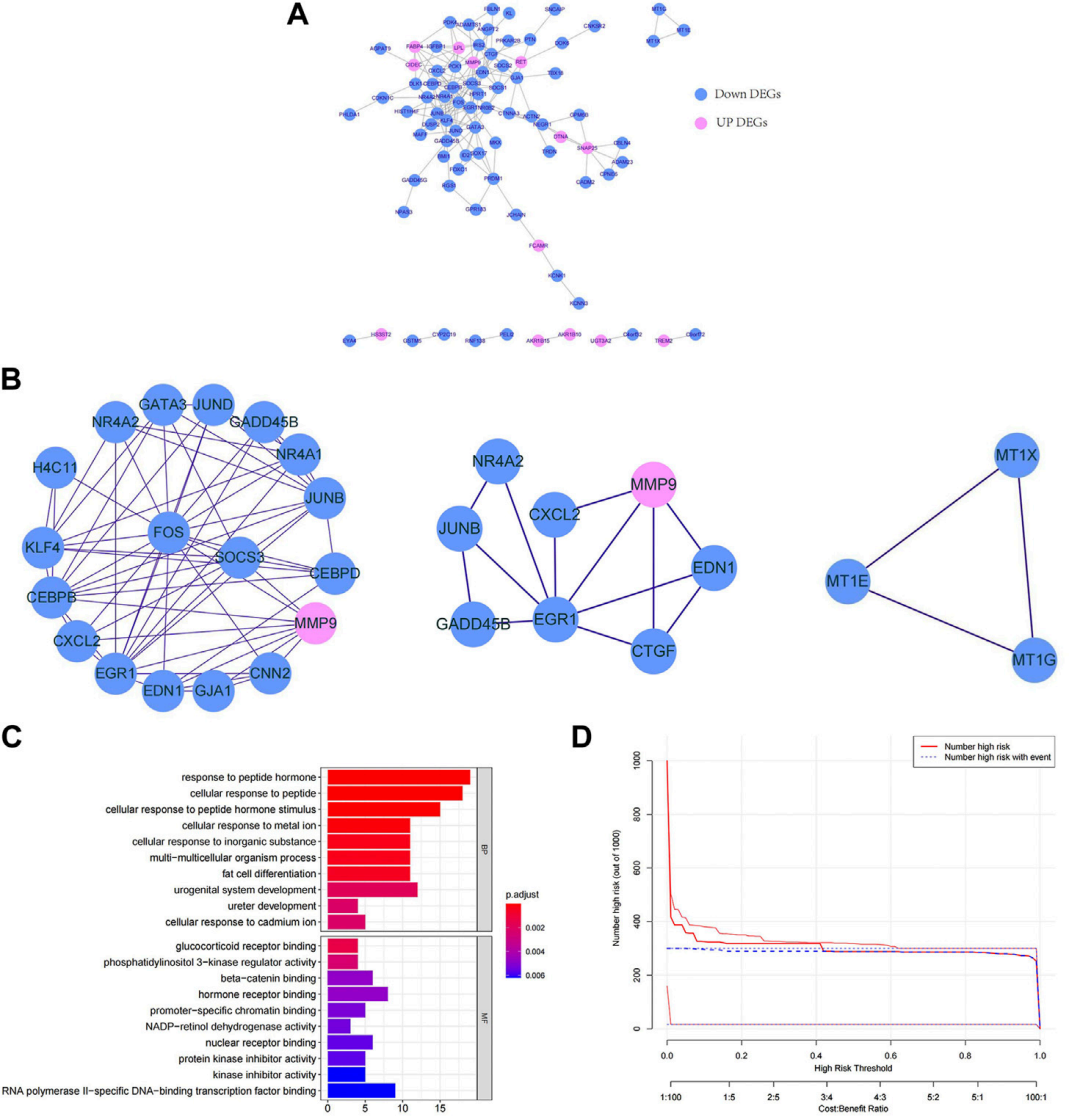
Functional enrichment analysis and interaction of key DEGs. **(A)** PPI network of key DEGs. **(B)** Key modules in the PPI network. The lines indicate the interaction between the DEGs. **(C)** GO enrichment bars for key DEGs. The ordinate indicates the enriched GO term, the length of the bars indicates the number of DEGs, and the colour from blue to red indicates the confidence from low to high. **(D)** Bubble chart of KEGG functional enrichment for key DEGs. The ordinate indicates the enrichment KEGG pathway, the bubble size indicates the number of DEGs, and the colour from blue to red indicates the confidence from low to high.

To explore the interactions among the 147 key DEGs, a PPI network of 147 genes was constructed. After removing the discrete proteins, 87 nodes and 360 edges were obtained. Cytoscape was used to visualize the interactive relationship network, as shown in Figure 2A. Moreover, a total of 3 key modules were obtained, and module 1 included CEBPB, H4C11, JUND, SOCS3, FOS, CEBPD, KLF4, GATA3, and NR4A1. Module 2 included GADD45B, JUNB, EGR1, NR4A2, CXCL2, EDN1, CNN2, and MMP9. Module 3 included MT1G, MT1E, and MT1X (Figure 2B). Module 1 was regarded as the best module based on the maximum score. Therefore, genes in module 1 were selected for subsequent analysis.

Next, we further explored the targeted pathways and functions of the 147 key DEGs. As shown in Figure 2C, key DEGs were significantly related to the response to abiotic stimuli, ureter development, adipocyte differentiation, etc. For molecular functions, key DEGs were significantly related to receptor binding and protein kinase inhibitor activity. Notably, the genes of module 1 were significantly related to osteoblast differentiation and positive regulation of ossification. The genes of module 2 were significantly associated with kidney development, response to oxygen level and response to metal ions. The genes of module 3 were significantly involved in the reaction to metal ions and the interpretation of inorganic compounds. KEGG functional enrichment analysis revealed that key DEGs were mainly involved in auxin synthesis, parathyroid hormone synthesis, osteoclast differentiation and insulin signal transduction (Figure 2D). The genes of module 1 and module 2 were mainly associated with the IL-17 and TNF signalling pathways. The genes in module 3 were associated with mineral absorption.

### Machine learning screening for diagnostic biomarkers

**FIGURE 3:**
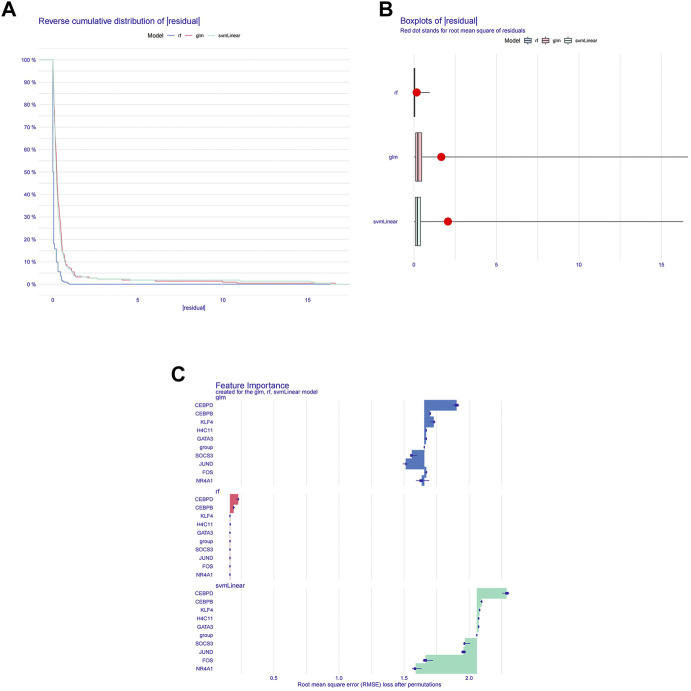
Machine learning screening for diagnostic biomarkers. **(A)** Distribution graphs of sample cumulative residuals. The area under the curve indicates the cumulative residual value of all samples. **(B)** Boxplot of sample residuals. Red dots indicate the root mean square. **(C)** Importance of gene variables in the RF, GLM, and SVM models.

To further screen diagnostic markers from the genes in module 1, three machine algorithms were used to construct three models separately. The RF model was the most suitable model because it had the smallest sample residual (Figures 3A,B). Moreover, as shown in Figure 3C, the five variables CEBPD, H4C11, CEBPB, GATA3, and KLF4 in the RF model had a strong influence on the predicted value of the response variable, so these five genes were used as diagnostic biomarkers for further analysis.

### Construction and validation of a nomogram

**FIGURE 4:**
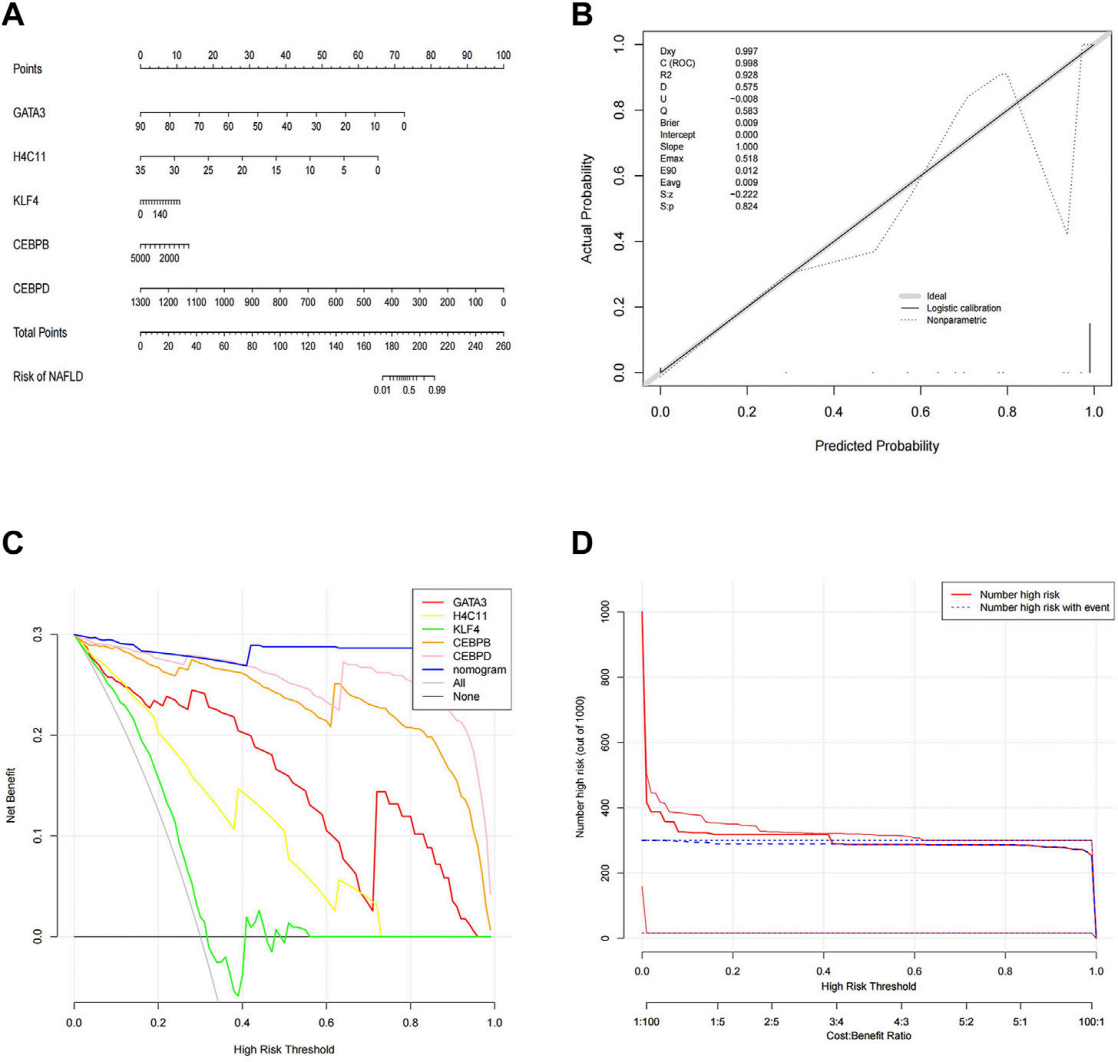
Construction and validation of a nomogram. **(A)** Nomogram of diagnostic biomarkers. **(B)** Calibration curves of the predictive nomogram model. **(C)** DCA curve to evaluate the clinical application value of the nomogram model. **(D)** Clinical impact curves of the nomogram model. The nomogram model predicts a risk stratification of 1,000 people. At each high-risk threshold, the number of high-risk curves indicates the number of models classified as positive (high risk), and the number of high-risk curves indicates the number of true positive people.

To better predict the risk of NAFLD by using CEBPD, H4C11, CEBPB, GATA3, and KLF4, a nomogram was constructed (Figure 4A). The nomogram was scored each biomarker. Then, the risk of NAFLD was predicted according to the total score. Moreover, calibration curves were drawn to verify the nomogram. Interestingly, calibration curves showed that the error between the actual and predicted risk of NAFLD was small, indicating that the nomogram model had a high prediction accuracy for NAFLD (Figure 4B). Furthermore, the DCA curve showed that the nomogram curve was higher than the grey line, “GATA3” curve, “H4C11” curve, “KLF4” curve, “CEBPB” curve and “CEBPD” curve (Figure 4C). The results showed that the nomogram model could benefit from a risk threshold range of 0–1, and the clinical benefit of the nomogram model was higher than that of the GATA3, H4C11, KLF4, CEBPB, and CEBPD curves. In the clinical impact curve (Figure 4D), from 0 to 1, the “Number High Risk” curve under the high-risk threshold was very close to the “Number High Risk with Event” curve, indicating that the nomogram model had a relatively accurate prediction ability.

### Correlation between diagnostic biomarkers and immune cell infiltration

**FIGURE 5:**
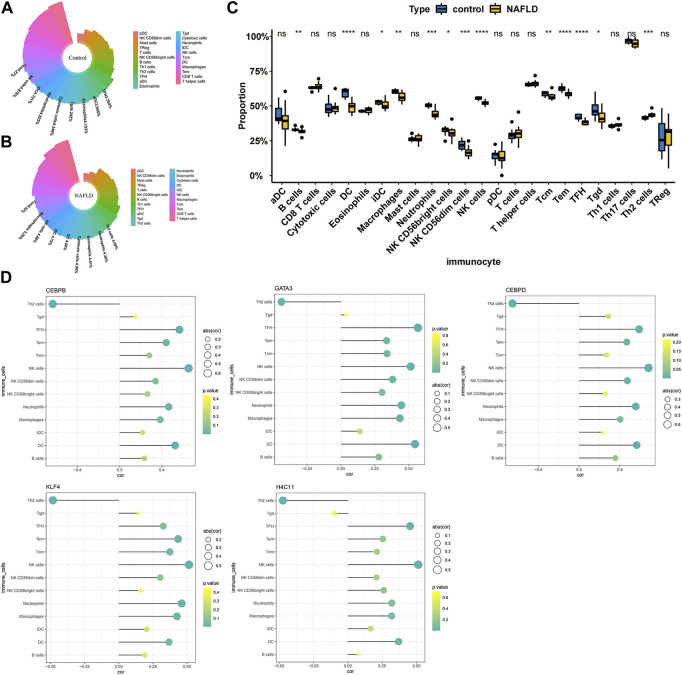
Correlation between diagnostic biomarkers and immune cell infiltration. **(A)** Nightingale rose diagram of immune cell proportions in the normal group samples. **(B)** Nightingale rose diagram of immune cell proportions in the NAFLD group samples. Each colour indicates an immune cell; the larger the area per colour, the larger the proportion of cells. **(C)** Boxplot of the immune cell proportion. “*”*p* < 0.05, “**”*p* < 0.01, “***”*p* < 0.001, and “****” *p* < 0.0001. **(D)** Lollipop chart of the correlation between diagnostic biomarkers and immune cells. The circle size indicates the correlation magnitude; the colour from green to yellow indicates an increased *p-value*.

To further explore the correlation between diagnostic biomarkers and immune cell infiltration, we compared the immune infiltration between patients with NAFLD and normal samples. The score of each immune cell in each sample was calculated by the ssGSEA algorithm. As shown in Figures 5A,B, the top five immune cells in the normal group were macrophages, TEM cells, CD8 T-cells, T helper cells, and DCs. In the NAFLD group, the top five immune cells were TCM, TEM, CD8 T-cells, T helper cells, and macrophages. In addition, a total of 13 types of immune cells, including DCs, NK cells, TFH cells, Tems, neutrophils, NK CD56dim cells, macrophages, Th2 cells, Tcm cells, B cells, Tgd cells, NK CD56bright cells, and iDCs, showed significant differences between NAFLD and normal samples (*p* < 0.05) (Figure 5C). Furthermore, Pearson correlation analysis between biomarkers and different immune cells showed that Th2 cells had a strong negative correlation with GATA3 (cor = −0.439), KLF4 (cor = −0.482), H4C11 (cor = −0.473), CEBPD (cor = −0.654), and CEBPB (cor = −0.634), while other immune cells showed a significant positive correlation with these biomarkers (Figure 5D).

### Potential drug prediction

**FIGURE 6:**
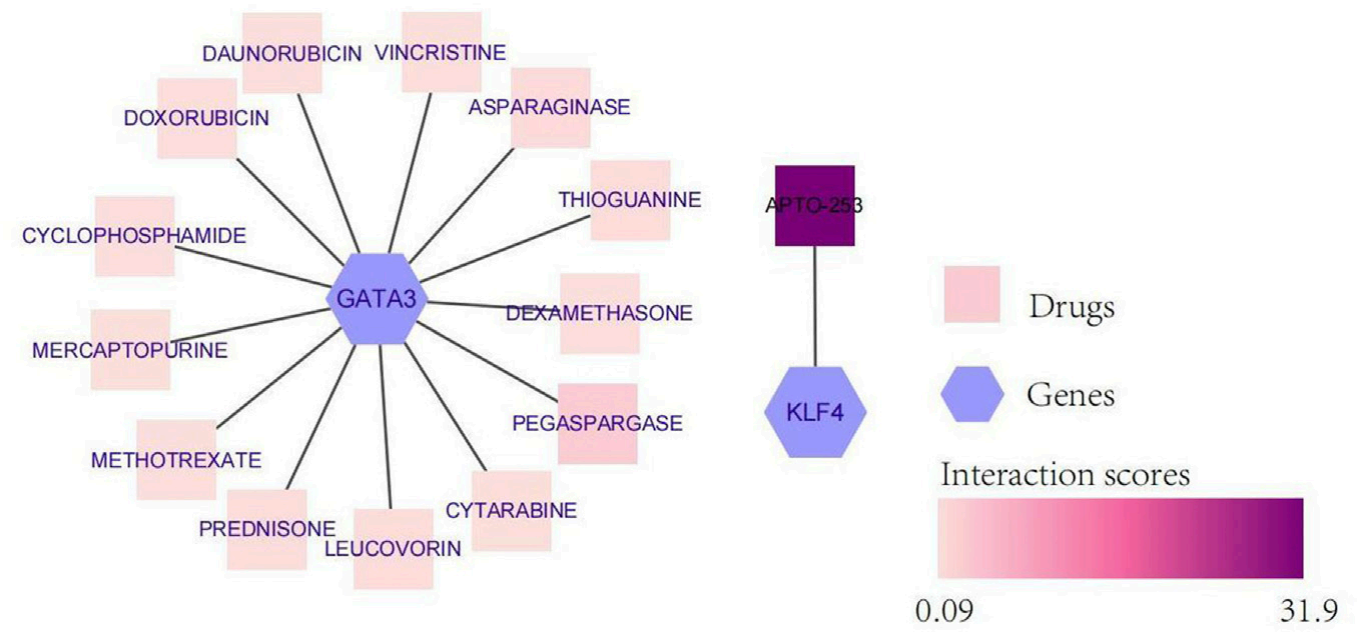
Network diagram of diagnostic biomarkers and potential therapeutic drugs. The hexagon indicates the biomarker, and the square indicates the potential therapeutic drug; the darker the potential therapeutic drug is, the higher the interaction score.

To explore potentially targeted therapeutic drugs that may be the most suitable for targeting diagnostic biomarkers, we retrieved 5 markers from the DGIdb database. Finally, we found two genes with related drugs. No drugs were found for the CEBPD, H4C11 and CEBPB genes. The potential therapeutic drugs predicted by GATA3 were pegaspargase, asparaginase, thioguanine, leucovorin, prednisone, mercaptopurine, cytarabine, vincrisine, daunorubicin, cyclophosphamide, dexamethasone, and methotrexate. The potential therapeutic drug predicted by KLF4 was APTO-253. The top three drugs were APTO-253, pegaspargase and asparaginase (Table 1; Figure 6).

**TABLE 1:** Potential therapeutic drugs corresponding to the diagnostic biomarkers.

Gene	Drug	Sources	PMIDs	Query score	Interaction score
GATA3	Pegaspargase	PharmGKB	24141364	2.19	2.45
GATA3	Asparaginase	PharmGKB	24141364	0.58	0.65
GATA3	Thioguanine	PharmGKB	24141364	0.34	0.38
GATA3	Leucovorin	PharmGKB	24141364	0.31	0.35
GATA3	Prednisone	PharmGKB	24141364	0.27	0.31
GATA3	Mercaptopurine	PharmGKB	24141364	0.24	0.27
GATA3	Cytarabine	PharmGKB	24141364	0.2	0.23
GATA3	Vincristine	PharmGKB	24141364	0.15	0.17
GATA3	Daunorubicin	PharmGKB	24141364	0.13	0.15
GATA3	Cyclophosphamide	PharmGKB	24141364	0.11	0.12
GATA3	Dexamethasone	PharmGKB	24141364	0.1	0.12
GATA3	Doxorubicin	PharmGKB	24141364	0.09	0.1
GATA3	Methotrexate	PharmGKB	24141364	0.08	0.09
KLF4	APTO-253	TTD	None found	2.19	31.9

### Validation of the expression of biomarkers

**FIGURE 7:**
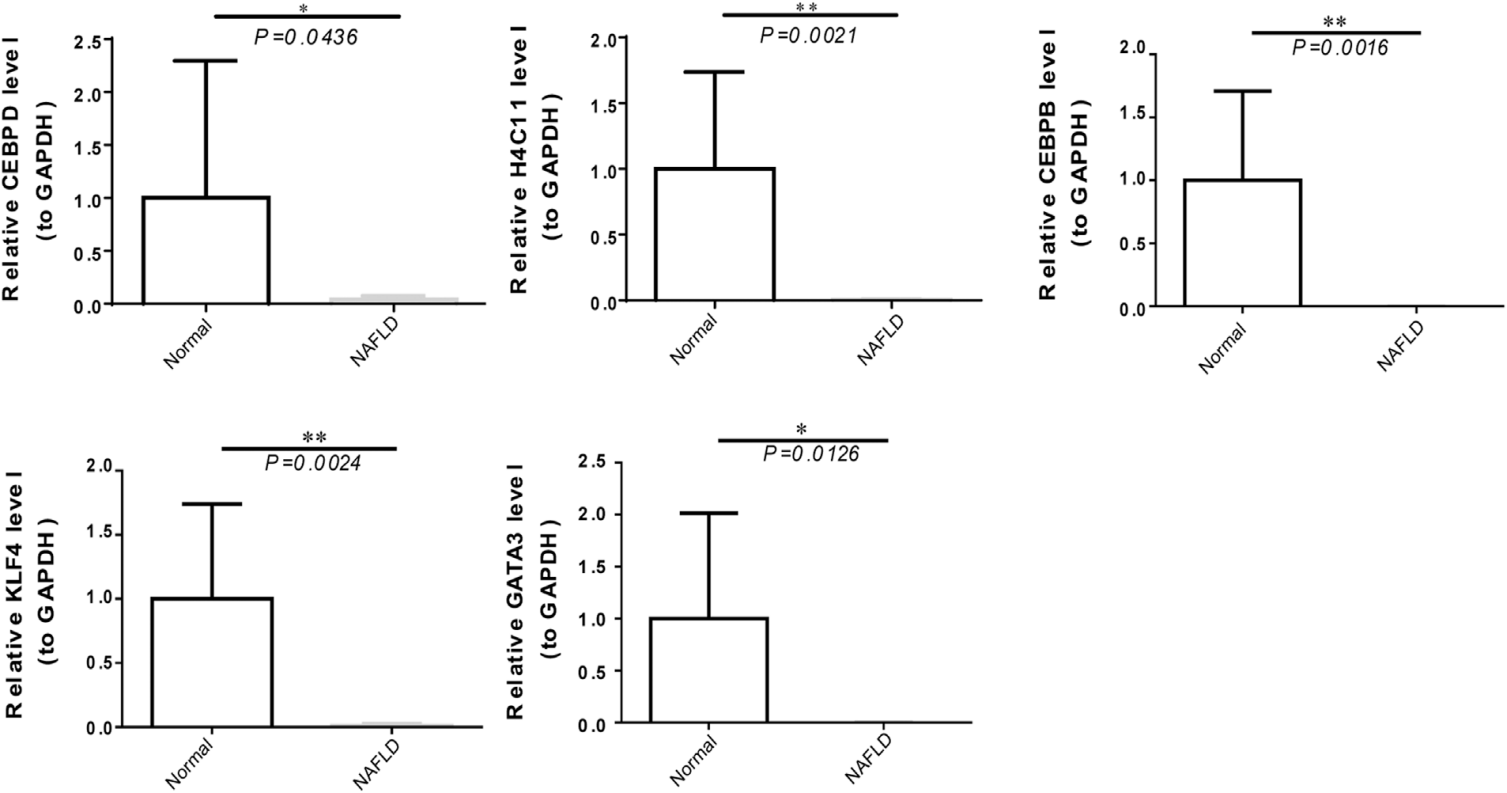
The expression of diagnostic biomarkers in the peripheral blood. Verification of CEBPD, H4C11, CEBPB, GATA3, and KLF4 in peripheral blood mononuclear cells of normal controls and NAFLD patients *via* qRT‒PCR.

To further verify the expression of biomarkers, we used qRT‒PCR to compare the gene expression levels of CEBPD, H4C11, CEBPB, KLF4, and GATA3 in the peripheral blood of normal controls and NAFLD patients. The qRT‒PCR results showed significant downregulation of the expression of CEBPD, H4C11, CEBPB, KLF4, and GATA3 in NAFLD patients (Figure 7).

## Discussion

NAFLD is a liver disease associated with obesity, insulin resistance, type 2 diabetes mellitus (T2DM), hypertension, hyperlipidaemia, and metabolic syndrome (Younossi, 2019; Lazarus et al., 2022b; Adams et al., 2017; Anstee et al., 2013). The pathogenesis of NAFLD is still unclear, and the “two-hit” hypothesis has been proposed. The first hit is elevated hepatic lipid accumulation caused by insulin resistance. Due to the first hit, the liver becomes more sensitive to a second hit, such as oxidative stress, lipid peroxidation and inflammation. Although it is primarily a disease of disturbed metabolism, NAFLD involves several immune cell-mediated inflammatory processes, particularly when reaching the stage of NASH, at which point inflammation becomes integral to the progression of the disease (Huby and Gautier, 2021). Sentinel cells in the liver sense excess metabolites, damaged hepatocytes and bacterial products and translate those signals into immune responses, resulting in steatohepatitis (Nati et al., 2022). Inflammation in the context of fatty liver is not a one-way route towards progression but rather a tug of war between necroinflammation and phases of resolution. Excess nutrients lead to the accumulation of fat and hypertrophy of adipose tissue. This initiates an immune response with the recruitment of proinflammatory cells (Peiseler and Tacke, 2021). Gut-derived LPS induces inflammatory pathways in adipose tissue through TLR4 signalling, enhancing the recruitment of proinflammatory monocytes (Caesar et al., 2015). Therefore, exploration of the role of immune cells in all stages of NAFLD can provide new strategies for the prevention and treatment of NAFLD.

In our study, the diagnostic biomarkers identified by transcriptomic analysis were differentially expressed in 13 types of immune cells. CEBPB, CEBPD, GATA3, KLF4, and H4C11 were the genes identified by our transcriptomics analysis of the NAFLD samples, which were primarily involved in immune cells and had been identified as a target by several disease studies.

CEBPB and CEBPD are CCAAT/enhancer-binding protein beta and delta, important transcription factors regulating the expression of genes involved in immune and inflammatory responses. CEBPB and CEBPD have been confirmed to have transcriptional activity in the inflammatory response, and the current work showed that their downregulation was associated with the loss of immune-related signals (Liu et al., 2019). CEBPB and CEBPD are activated by inflammatory factors in inflammatory environments (Cantwell et al., 1998) (Tengku-Muhammad et al., 2000). Meanwhile, CEBPB and CEBPD regulate preadipocyte differentiation and participate in lipid metabolism by activating PPARγ. Moreover, CEBPB and CEBPD might promote NAFLD through inflammatory activation of the liver and lipid metabolism, but the specific mechanisms still need to be explored further.

GATA3 belongs to a family of transcription factors and is generally thought to play important roles in haematopoiesis, nervous system development (Lowry and Atchley, 2000) (Patient and McGhee, 2002), and inflammatory and humoral immune responses (Ray and Cohn, 1999; Wan, 2014). Regarding immunoregulation, GATA3 was originally identified as a master regulator of Th2 cell differentiation of CD4^+^ T-cells. It is also critical for the development, differentiation, and function of other CD4^+^ T-cell subsets, as well as CD8^+^ cells. GATA3 controls the function of both adaptive and innate immune cells. Recent findings conclude that although GATA3 allows Th17 cell differentiation, it acts as an inhibitor of Th17-mediated pathology, through IL-4-dependent and IL-4-independent pathways (van Hamburg et al., 2008). Meantime, IL-17 secreted mainly by Th17 cells is a key cytokine involved in NAFLD (Gomes et al., 2016) and atherosclerosis following obesity-related NAFLD (Tarantino et al., 2014). Indeed, GATA3 is also expressed in many cells in adipose tissues, including preadipocytes, mature adipocytes, and various inflammatory cells. GATA3 plays an important role in adipogenesis (Al-Jaber et al., 2021). GATA3 suppresses adipocyte differentiation partially through direct binding to peroxisome proliferator-activated receptor *γ*. It also forms protein complexes with CEBPB, and this interaction subsequently suppresses adipocyte differentiation (Tong et al., 2005) (Tong et al., 2000). Our study showed that GATA3 and CEBPB were negatively correlated with Th2 cells in liver tissue, which is consistent with previous studies. Therefore, we infer that GATA3 may be involved in the progression of NAFLD by regulating the natural immune signalling pathways of the liver and producing a variety of inflammatory and lipid metabolism effector molecules.

KLF4, an important transcription factor of the KLF family, and it has been proven to be related to biological processes related to cellular proliferation, differentiation, and self-renewal (Alder et al., 2008) (Liao et al., 2011b). Current studies have shown that KLF4 has many roles, such as inhibiting and promoting tumour progression, regulating the cell cycle, influencing macrophage polarization, regulating the inflammatory response, and affecting atherosclerosis. Studies have shown that KLF4 cooperates with Stat6 to induce an M2 macrophage genetic program and inhibit M1 macrophage targets *via* sequestration of coactivators required for NF-κB activation (Han et al., 2017). Moreover, patients with simple steatosis had higher levels of M2 macrophages in the liver than patients with severe steatohepatitis (Liao et al., 2011a). The regulation of M1/M2 polarization in liver macrophages is associated with the progression of NASH. The M2-promoting effects of KLF4 in liver macrophages may provide better therapeutic strategies against NASH.

H4C11 is one of the histones responsible for the nucleosome structure of chromosomal fibres in eukaryotes (Rabdano et al., 2021). Histone H4 participates in the initiation of DNA template transcription and negatively regulates megakaryocyte differentiation. Studies have shown that histone H4 could be used as a molecular target for antiaging drug screening, research and development (Lin et al., 2020). Histone modifications consist of acetylation, methylation, phosphorylation, and ubiquitylation. Among them, histone acetylation patterns are the most studied pattern. They are known to be regulated by histone acetyltransferases and histone deacetylases (Fu et al., 2021). Accumulating evidence has shown that histone deacetylation is involved in the metabolic mechanism and pathogenesis of diseases, including NAFLD (Tian et al., 2015). However, the role of histone modifications in NAFLD has not yet been explored.

Finally, we retrieved five diagnostic biomarkers from the DGIdb database and obtained potential drugs associated with GATA3 and KLF4 for the treatment of NAFLD. Among them, GATA3 predicted multiple-targeted drugs (as shown above), which have been shown to increase the incidence of fatty liver disease during or after treatment. Studies have shown that some drugs activate PPARα, leading to lipolysis and fatty acid oxidation in adipose tissue and increasing the circulating fatty acid level and their transfer to the liver, resulting in disorders of PPARγ and ApoB, further insulin resistance and hepatic steatosis (Renu et al., 2019) (Ben-Yakov et al., 2019) (for the Drug-Induced Liver Injury Network et al., 2019). It was predicted by KLF-4 that APTO-253 could be a targeted therapeutic agent for NAFLD. A previous study revealed that the KLF4-NOXA axis was involved in the induction of p53-independent apoptosis in response to DNA damage (Nakajima et al., 2021). In addition, induction of KLF4 in macrophages could promote the proinflammatory M1 to anti-inflammatory M2 phenotype by a STAT6-dependent mechanism. Whether APTO-253, as a KLF4 activator, can induce polarization in macrophages needs to be confirmed by further research.

## Conclusion

In conclusion, CEBPD, H4C11, CEBPB, GATA3, and KLF4 were identified as diagnostic biomarkers of NAFLD by machine learning algorithms and were related to immune cell infiltration in NAFLD. These key genes can help us more deeply understand the pathogenesis of NAFLD.

## Data Availability

The datasets presented in this study can be found in online repositories. The names of the repository/repositories and accession number(s) can be found in the article/Supplementary Material.
